# Hydrophobicity-Tuned Periodic Mesoporous Organo-Silica Nanoparticles for Photodynamic Therapy

**DOI:** 10.3390/ijms21072586

**Published:** 2020-04-08

**Authors:** Chia-Hui Lin, Ranjith Kumar Kankala, Prabhakar Busa, Chia-Hung Lee

**Affiliations:** 1 Department of Life Science, National Dong Hwa University, Hualien 97401, Taiwan; m9913123@gms.ndhu.edu.tw (C.-H.L.); ranjithkankala@hqu.edu.cn (R.K.K.); prabhakar.busa01@gmail.com (P.B.); 2 College of Chemical Engineering, Huaqiao University, Xiamen 361021, China

**Keywords:** periodic mesoporous organosilicas, nanotechnology, Fenton-like reaction, photodynamic therapy, anti-cancer, anti-bacterial

## Abstract

Since their invention, periodic mesoporous organosilicas (PMOs), an innovative class of materials based on organic as well as inorganic hybrid nanocomposites, have gathered enormous interest owing to their advantageous physicochemical attributes over the pristine mesoporous silica nanoparticles (MSNs). To further increase the interactions with the therapeutic guest species and subsequent compatibility as well as the physicochemical properties of PMOs, we demonstrate the post-hydroxylation of benzene-bridged PMO-based nanoparticles for photodynamic therapy (PDT). Initially, the hydrophobic benzene group in the PMO framework is modified through electrophilic substitution-assisted hydroxylation mediated by Fenton as well as Fenton-like reactions utilizing divalent and trivalent metal salts, respectively. These post-grafted PMOs with tuned hydrophobicity resulted in improved biocompatibility as well as drug loading efficiency through governing the interactions in host–guest chemistry by changing the physicochemical properties of the PMO frameworks. Furthermore, the photosensitizer, protoporphyrin IX (PpIX) molecules, encapsulated in the PMO frameworks showed a significant PDT effect in colon carcinoma (HT-29 cell line) and Gram-negative bacterial strain, *Escherichia coli (E. coli)*. Furthermore, the light-induced cytotoxic properties in vitro are confirmed by various tests, including lactate dehydrogenase (LDH) assay for cell membrane damage and caspase assay for apoptosis determination. Indeed, the delivered PpIX molecules from PMOs generated deadly singlet oxygen species intracellularly under visible light irradiation, resulting in cell death through concomitantly triggered apoptotic caspases. Together, our findings demonstrate that this post-modified PMO design is highly advantageous and can be used as an effective PDT platform.

## 1. Introduction

In recent times, the fabrication of nanomaterials and enormous advancements in improving their inherent characteristics have continued to rise towards their potential utility in the field of nanomedicine [1]. Amongst various inorganic-based nanomaterials, mesoporous silica nanoparticles (MSNs) have gathered immense attention owing to their highly unique physicochemical properties, such as a large specific surface area, extensive pore volume with tunable pore sizes, and ease of surface functionalization for loading various guest molecules as well as controllable particle sizes and shapes, among others [2,3]. These highly impressive physicochemical characteristics, as well as morphological attributes along with the provision of stability to the guest species in the harsh biological environments, biocompatibility, as well as considerable biodegradability in the physiological fluids, are of specific interest to various biomedical applications, such as controlled and targeted drug delivery, imaging, and tissue engineering, among others [2,4,5,6,7,8]. However, the comprehensive biological behavior and controlled degradation of silica framework (Si-O-Si) and its clinical performance remain inherent challenges.

Since the invention of mesoporous silica-based materials by Mobil group scientists, tremendous advancements utilizing MSNs over the past few decades have evidenced the fabrication of various innovative composites for various biomedical applications [2,5,9,10]. Along this line, parallel efforts by Asefa and coworkers resulted in a new class of mesostructured materials by incorporating organic components in the siliceous frameworks to improve the chemical functionalities and biodegradability of MSNs [11]. These organo-bridged silsesquioxane ((R′O)_3_Si-R-Si(R′O)_3_)-integrated mesoporous silica frameworks, often denoted as periodic mesoporous organosilica (PMOs), offer enormous advantages. One such advantage is the provision of true active sites for subsequent surface functionalization towards guiding the encapsulation of guest species [10,12,13,14,15,16]. These innovative organo-bridged inorganic nanocomposites greatly changed the MSN characteristics, including enhanced physicochemical attributes and biocompatible properties, by decreasing the siloxane contact [17]. In addition, these organic bridging groups can establish interactions for the stimuli-responsive release of biomolecules without any capping/gates to the mesopores [18]. More often, the synthesis of PMOs is preceded by the hydrolysis and subsequent co-condensation of single organic group-bridged organo-siloxane similar to traditional MSNs utilizing silica precursors, such as tetramethoxysilane (TMOS) or tetraethoxysilane (TEOS) [19]. Along this line, a variety of PMOs have been fabricated using various silanes containing different organic moieties, viz. phenyl [20,21], thiophene [21], biphenyl [22], divinylbenzene [23], 2,20-bipyridine [24], and bis-imidazolium [25], among others [9,19,26,27]. Although these organic moieties in the framework allow the encapsulation of the guest species, the post-modification of the organic bridging moiety significantly enhances the performance characteristics of PMOs through fine-tuning the hydrophobic character, which improves the host–guest chemistry interactions and govern the physicochemical properties of the framework. For example, the post-synthesis bromination of benzene-bridged PMOs considerably eliminated its unwanted structural deterioration and potential for the structural and functional improvements [12].

In recent times, PMOs have been used for the fabrication of delivery vehicles towards light-assisted therapeutics [19,28,29,30,31]. In an instance, Durand and coworkers prepared dual-functionalized ethenylene-based PMOs for two-photon imaging as well as photodynamic therapy (PDT) [32]. In another case, they demonstrated the synthesis of nanodiamond–1,2-bis(triethoxysilyl)ethane-based PMOs as core-shell platforms for two-photon PDT through the pH-sensitive release of doxorubicin [33]. Similarly, Zhao and colleagues synthesized core-shell PMOs by anisotropically growing the organosilica framework on upconversion nanoparticle using 1,2-bis(triethoxysilyl)-ethane as an organosilane source for the near-infrared (NIR) light-irradiated bimodal-triggered release of dual drugs [28]. In another approach, a nanoporous hybrid material featuring a fluorescent moiety of tris(propyliminomethyl)-phloroglucinol in MSNs was prepared for the targeted delivery of doxorubicin [34]. In another study, Wang and colleagues synthesized uniform yolk-shell triple-hybridized PMOs simultaneously incorporated with various organic (ethane, benzene, and thioether)-bridging units in the mesoporous siliceous framework, which resulted in exceptional biocompatibility and drug loading capability [35]. Interestingly, for the development of anti-cancer modalities, Chen and coworkers synthesized benzene-bridged hollow PMOs for high intensity-mediated ultrasound-triggered drug release towards synergistic cancer therapy [36].

Inspired by these aforementioned facts and considerations, herein, we demonstrate the hydroxylation of benzene-bridged PMO nanoconjugates for improved PDT efficacy. To increase the interactions with the therapeutic guest species and subsequent compatibility as well as the stability attributes of PMOs, the hydrophobic benzene group in the PMO framework is modified by installing the hydroxyl groups mediated by Fenton as well as Fenton-like reactions utilizing divalent and trivalent metal salts, respectively. Finally, the protoporphyrin IX (PpIX)-encapsulated PMOs (PMO-PpIX) were tested for the light-induced metabolic inactivation of tumor cells and bacteria under the visible light irradiation using the green laser light at 532 nm.

## 2. Results and Discussions

**Figure 1:**
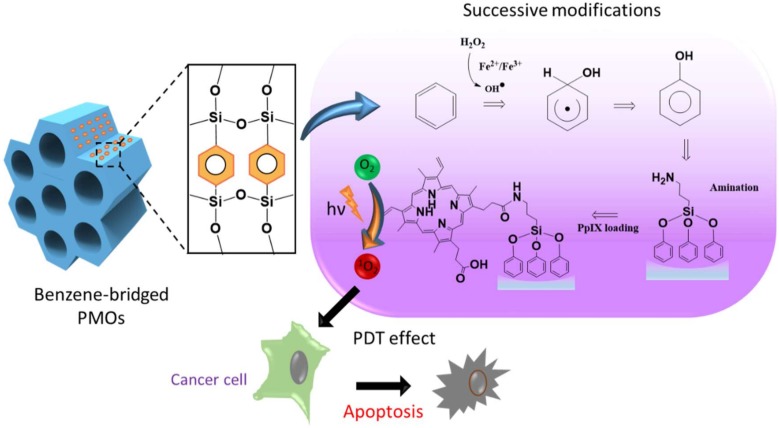
Schematic representing the structure of the periodic mesoporous organosilica (PMO) framework, the reaction pathway of the hydroxylation of benzene and protoporphyrin IX (PpIX) loading, and the mechanism elucidating the light-induced biological effects.

Initially, we synthesized well-ordered, benzene-bridged PMO nanocomposites. Furthermore, the host–guest chemistry in PMOs was improved through governing the interactions by altering their physicochemical properties. To achieve this, the hydrophobicity of the organic-moiety bridged PMO frameworks was tuned by the post-synthesis hydroxylation of benzene through the electrophilic substitution reaction in the presence of a hydroxyl group (-OH) donor, hydrogen peroxide (H_2_O_2_) (Figure 1). In this framework, the hydroxylation of benzene was performed in two ways, i.e., Fenton (Equation (1)) and Fenton-like reactions (Equation (2)) using ferrous sulfate (Fe(II)SO_4_) and ferric chloride (Fe(III)Cl_3_), respectively. The generated respective conjugates were denoted as PMO-OH(II) and PMO-OH(III) (Equation (3)).

Fe^2+^ + H_2_O_2_ → Fe^3+^ + HO. + OH^−^ (1)

Fe^3+^ + H_2_O_2_ → Fe^2+^ + HOO. + H^+^ (2)

C_6_H_6_ + Fe^2+^/Fe^3+^ + H_2_O_2_ → C_6_H_6_OH + Fe^3+^/Fe^2+^ (3)

Furthermore, these hydroxyl group-immobilized PMOs were grafted with the amine functional group, resulting in corresponding PMO-OH(II)-NH_2_ and PMO-OH(III)-NH_2_ samples. These modifications can enhance the biocompatibility as well as the stability of PMOs. On the other hand, to evaluate the post-modification efficiency, another batch of naked PMOs was grafted with the amine groups, and the sample is denoted as PMO-NH_2_. Further, the photosensitizer PpIX was pre-modified to improve the hydrophilicity and then immobilized in the optimized PMOs sample. Then, we report the PDT efficacy measurements, such as anti-cancer activity through 3-(4, 5-dimethylthiazol-2-yl)-2, 5-diphenyltetrazolium bromide (MTT) assay and the plausible mechanistic way through lactate dehydrogenase (LDH) and caspase-3-based assays for apoptosis confirmation. In addition, the antibacterial efficacy, through colony-forming unit (CFU) assay, was determined.

### 2.1. Characterizations

**Figure 2:**
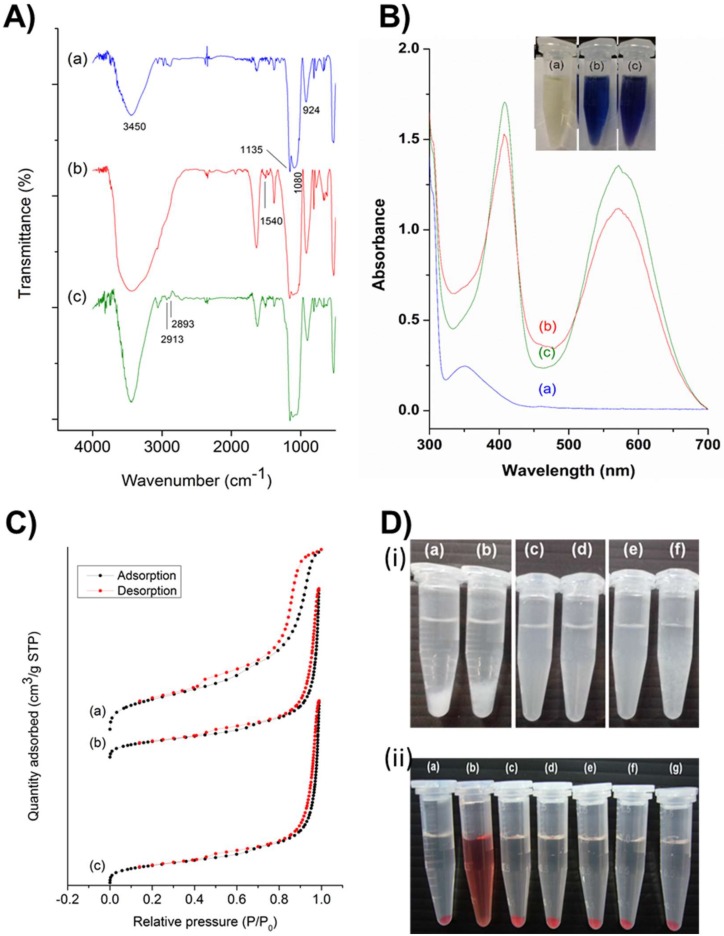
(**A**) Fourier transform infrared (FT-IR) spectra of (**a**) PMO, (**b**) PMO-OH(II)-NH_2_, and (**c**) PMO-OH(III)-NH_2_. (**B**) Ninhydrin tests of different post-modified PMO nanoconjugates showing the absorption spectra using the ultraviolet-visible (UV-vis) spectrophotometer and the inset figure showing the respective photographs of the sample tubes. (**a**) Ninhydrin alone, and in reaction with (**b**) PMO-OH(II)-NH_2_, and (**c**) PMO-OH(III)-NH_2_. (**C**) Nitrogen adsorption–desorption isotherm curves of various PMOs. Black dots represent the adsorption isotherm curves, and red dots represent the desorption isotherm curves. (**a**) PMO, (**b**) PMO-OH(II)-NH_2_, and (**c**) PMO-OH(III)-NH_2_. (**D**) (**i**) The stability test of PMOs and its successive conjugates (1 mg/mL) suspended in PBS. (**a**) PMO, (**b**) PMO-NH_2_, (**c**) PMO-OH(II), (**d**) PMO-OH(II)-NH_2_, (**e**) PMO-OH(III), and (**f**) PMO-OH(III)-NH_2_. (**ii**) Hemolysis test of optimized PMO sample in Red blood cells (RBCs). (**a**) Negative control (PBS), (**b**) Positive control (dd-H_2_O), (**c**) 1600, (**d**) 800, (**e**) 400, (**f**) 200, and (**g**) 100 µg/mL of PMO-OH(II)-NH_2_.

The synthesized PMO-based nanoensembles and their successive conjugates were characterized systematically by various techniques. Figure 2A depicts the Fourier transform infrared (FT-IR) spectra characterizing the robustness of the vibration scenario of the PMO framework and its post-synthetic modifications. A relatively broad absorption peak centered at around 3450 cm^-1^ could be attributed to O-H stretch, and an absorption peak at 924 cm^−1^ could be attributed to the O-H bending vibration of surface silanol groups and absorbed water molecules (Figure 2A-a). A robust and sharp absorption peak at 1135 cm^−1^ could be ascribed to the vibrations of ρ-substituted benzene-bridged silsesquioxane (-Si-O-C_6_H_4_-O-Si-). The relatively broad absorption peak at 1080 cm^−1^ ascribed to Si-O-Si asymmetric vibration on a siliceous framework skeleton. Figure 2A-b, c showed a new peak at 1540 cm^−1^, which could be attributed to N-H stretch, confirming the successful grafting of amine groups in the PMO framework. Furthermore, some peaks at 2913 and 2893 cm^−1^ could be attributed to the C-H stretch of the propyl group in the terminal amine chain. These shreds of evidence clarified that the PMO frameworks were modified successfully.

Furthermore, the post-modified amine functional groups were confirmed using the ninhydrin test. Figure 2B illustrates the ultraviolet-visible (UV-vis) absorption spectra of the amine-grafted PMO samples after being subjected to the ninhydrin test. Both the samples, i.e., PMO-OH(II)-NH_2_ (Figure 2B-b) and PMO-OH(III)-NH_2_ (Figure 2B-c), displayed the characteristic absorption peak of Ruhemann’s Purple at 405 and 570 nm. Moreover, the peak at 350 nm from the ninhydrin reactant disappeared, confirming the presence of amine functional groups in nanoparticle surfaces. Furthermore, it was evident from the inset figure that the tubes displayed positive confirmation of Ruhemann’s purple coloration after heating for ninhydrin-assisted amine-grafted PMOs, while the ninhydrin alone remained the same as light yellow. The dynamic light scattering (DLS) measurements revealed the particle size distribution, showing hydrodynamic diameters as well as zeta potential values of PMOs and its successive conjugates (Table 1). The hydrodynamic diameters of all nanoparticles, PMOs, PMO-OH(II)-NH_2_, and PMO-OH(III)-NH_2_ were in the range of around 100 nm (i.e., 106.1, 94.8, and 99.4 nm, respectively) and suspended well enough. Such hydrodynamic sizes are often conducive for exceptional stability and substantial cell internalization efficiency of nanoconjugates. The surface charge of the aqueous dispersion of PMOs and their successive conjugates was determined by dispersing the particles in water and adjusting the pH value to 7.0 (Table 1). Since MSNs typically possess a high negative charge, the surfactant-extracted PMOs resulted in -6.6 mV due to the high occupancy of benzene units. After the hydroxylation of PMOs, the zeta potential of resultant samples further increased towards negative values, i.e., PMO-OH(II) −13.1 mV and PMO-OH(III) −12.68 mV. Moreover, the zeta potential shifted towards the positive end after grafting amine functional groups (PMO-OH(II)-NH_2_ −5.7 mV, and PMO-OH(III)-NH_2_ −4.9 mV). These changes in the overall surface charge confirmed the immobilization of hydroxyl and amine functional groups in the PMO frameworks.

Furthermore, the mesoporous structural characteristics of PMOs and their modified nanoconjugates were demonstrated using nitrogen (N_2_) adsorption−desorption isotherms (Figure 2C), and their respective pore size distributions were calculated based on the Barrett–Joyner–Halenda (BJH) method (Table 1). The capillary condensation, evidenced by relative pressure (P/P_0_), was higher than 0.4. Moreover, no significant differences in the surface area and pore volume of various post-modified PMO nanoparticles in comparison to the surfactant-extracted naked PMOs were observed. These textural properties of surfactant-extracted benzene-bridged PMOs resulted in the surface area of 613 m^2^/g and their successive post-modified samples, PMO-OH(II)-NH_2_ and PMO-OH(III)-NH_2_ of 635 and 672 m^2^/g, respectively. Moreover, the average pore size and pore volume of PMOs were 7.23 nm and 2.06 cm^3^/g, respectively. At the same time, the samples (PMO-O(II)-NH_2_ and PMO-OH(III)-NH_2_) resulted in respective mesoporous confinement similar to the surface area.

### 2.2. Stability and Biocompatibility

Furthermore, the colloidal stability in PBS and biocompatibility by hemolysis assay of various PMO nanoconjugates were demonstrated. Despite the significant advancements in the delivery strategies and progress in biodegradability, PMOs still face difficulty in attaining excellent suspension stability in the aqueous environment. To evaluate the stability of designed PMO nanoconjugates, we performed stability tests by suspending the various nanoconjugates in PBS. It was observed from the experimental results that the PMOs (Figure 2D-i-a) and PMO-NH_2_ (Figure 2D-i-b) samples deposited more rapidly compared to those of the PMO samples after post-modification with OH functional groups, PMO-OH(II) (Figure 2D-i-c) and PMO-OH(III) (Figure 2D-i-e) and their corresponding amine-grafted samples (Figure 2D-i-d, f). Indeed, these consequences could be attributed to hydroxyl functionality, which increased the stability of suspensions effectively. Amongst these hydroxyl group-functionalized samples, PMO-OH(III)-NH_2_ has started to aggregate first compared to that of the PMO-OH(II)-NH_2_ suspension, which was still suspended well even after 30 min. Thus, the stable PMO-OH(II)-NH_2_ carrier was chosen as an optimal sample and used for further experiments, such as the immobilization of PpIX derivative and corresponding bioefficacy studies. Furthermore, the biocompatibility of these stable post-modified PMO samples (PMO-OH(II)-NH_2_) was demonstrated by the hemolysis test (Figure 2D-ii). This assay was performed by exposing various concentrations ranging from 100–1600 μg/mL of PMO-OH(II)-NH_2_ sample to red blood cells (RBCs) at room temperature for 3 h. Notably, the designed PMO nanoconjugates were highly biocompatible, which resulted in a clear supernatant and a lower hemolytic percentage (< 5%), signifying the safety profile. Contrarily, the membrane damage was observed in the positive control treatment, which resulted in the release of cell constituents, tissue hypoxia, and death. The interesting feature of these composites is that no signs of hemolysis were observed in RBCs treated with our design even at a high concentration of 1600 µg/mL. Henceforth, the designed PMO-OH(II)-NH_2_ nanoconjugates are highly biocompatible and could be suitable for theranostic nanomedicine.

### 2.3. Photobleaching Effect

**Figure 3:**
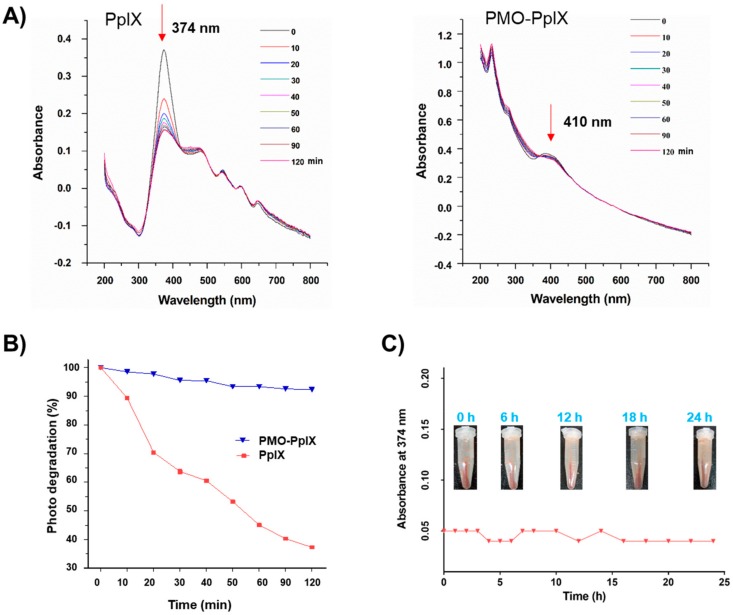
Photobleaching effect of PpIX. (**A**) UV-vis spectra at the normalized Soret band analysis of pure PpIX and PMO-PpIX formulation in aqueous solution in the presence of light irradiation at different time points. (**B**) The samples, free PpIX (λ_max_ at 374 nm), and PMO-PpIX (λ_max_ at 410 nm) were illuminated with a 532-nm green laser light, and the absorption intensities were recorded using the UV-vis spectrophotometer at the corresponding time intervals for 120 min. (**C**) UV-vis measurements showing the PpIX leakage from the designed PMOs in the biological buffer. Sample tubes in the inset showing the designed PMO-PpIX samples dispersed in RPMI-1640 medium (no phenol red) at corresponding time intervals for 24 h.

To demonstrate the ability of loaded photosensitizer, several investigations, such as the photobleaching effect for PpIX stability and the photo-induced singlet oxygen determination, were further performed. Considering the stability and compatibility attributes of the synthesized post-modified PMOs, the optimized Fenton reaction product (PMO-OH(II)-NH_2_) was chosen to graft the PpIX in the mesopore via hydrophilic interactions, and the sample was shortly denoted as PMO-PpIX. The PpIX loading efficiency was calculated as 10.8% by recording the absorbance of free PpIX in the supernatant using the UV-vis spectrophotometer at a wavelength of 374 nm. The loaded PpIX resulted in a significant shift in the absorbance at 410 nm, confirming its loading in the nanocontainers (Figure 3A). Notably, a shift in the characteristic peak of PpIX in the PMO-PpIX sample might be due to the grafted PpIX over the PMO surface through a covalent linkage. Furthermore, the photobleaching assay of the samples was performed to evaluate the stability of photosensitizer in the presence of light with respect to the exposure time. Compared to PMO-PpIX, the photobleaching rate of free PpIX decreased rapidly in 20 min after being illuminated with green laser light at 532 nm (Figure 3A,B). To this end, the photobleaching rate was attenuated to >90% at similar provided conditions, indicating that the photosensitizer molecules were safer after being immobilized in the stable PMO nanoconjugates over free PpIX. Indeed, it signified that PMOs could reduce the probability of active groups attacking PpIX and thereby safeguarded the photosensitizer from degradation in their confined spaces. In addition, we observed the leakage of PpIX from PMOs by dispersing them in the RPMI-1640 medium (no phenol red) for a specified time interval (Figure 3C). It was evident from the experimental results that no visible free PpIX color in the supernatant was observed with time, suggesting that the PpIX molecules were indeed grafted in the mesochannels of PMOs network through a stable linkage. 

**Figure 4:**
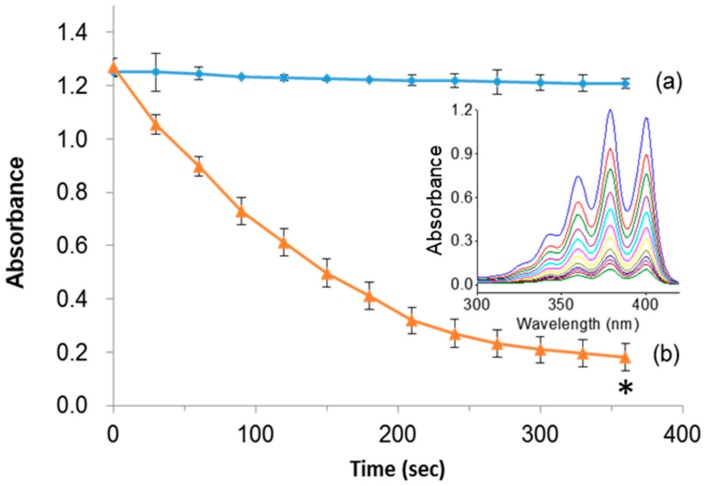
Photo-induced singlet oxygen detection. (**a**) 10-anthracenediyl-bis(methylene)dimalonic acid (ABMDMA) alone, and (**b**) ABMDMA in combination with PMO-PpIX. Inset figure showing the absorption spectra of (**b**) scanned at UV-vis wavelength range every 30 s. (* *p* < 0.001).

Indeed, the light-induced generation of deadly singlet oxygen (^1^O_2_) species plays a major role in ablating cancer cells. To demonstrate the singlet oxygen generation efficacy of PpIX, the designed PMO samples in the presence of light were tested by photo-induced degradation assay in vitro using the 9, 10-anthracenediyl-bis(methylene)dimalonic acid (ABMDMA) dye. The principle of photo-induced degradation by ABMDMA is that the dye interacts with singlet oxygen to form respective peroxide, resulting in reduced absorbance at 400 nm. Figure 4a shows that the ABMDMA dye alone, when illuminated with green laser light, resulted in no significant effect. Contrarily, the PMO-PpIX nanoconjugates, in combination with irradiated light, have shown a significant reduction in the absorption intensity of dye rapidly to half in the first 120 s, and 1/6th fraction in next 240 s (Figure 4b), indicating the generation of singlet oxygen species. Furthermore, the UV-vis absorption spectra confirmed the photobleaching effect of the ABMDMA dye in the presence of PMO-PpIX with respect to time, indicating the progressive reduction of absorption intensity corresponding to the increase in singlet oxygen generation with an increase in time of exposure (Figure 4b, inset).

### 2.4. PDT Effect in Cancer Cells

**Figure 5:**
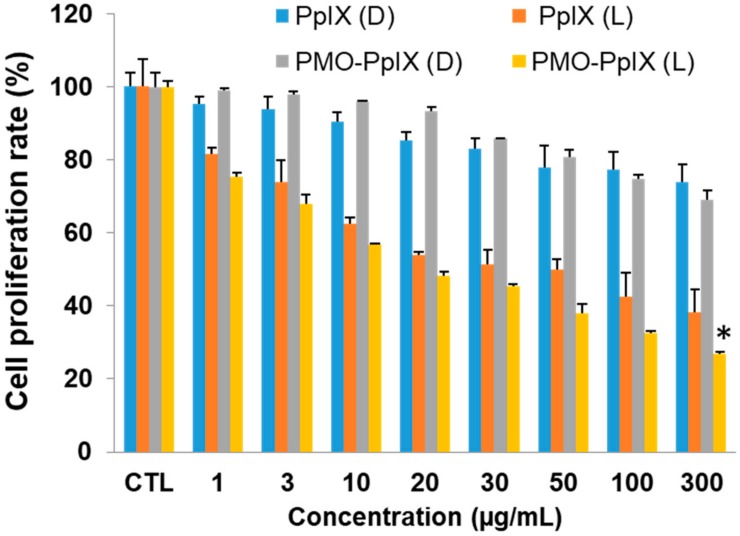
3-(4, 5-dimethylthiazol-2-yl)-2, 5-diphenyltetrazolium bromide (MTT) assay results showing the PDT effect of various concentrations of free PpIX and PMO-PpIX in HT-29 cells in the absence (D) and presence (L) of light irradiation. (* *p*< 0.001).

During PDT, the photosensitizers actively produce immense levels of toxic reactive oxygen species (ROS) (in this case, singlet oxygen by PpIX) under light irradiation, which, more often obstruct the cell metabolic activities, such as mitochondrial dysfunction and nucleus deterioration, among others. To evaluate the PDT effects of our design, we initially performed the cell proliferation studies in vitro of free PpIX and PMO-PpIX samples at various concentrations (*viz*. 0–300 µg/mL) in the presence and absence of light irradiation using MTT assay in the HT-29 cell line. Figure 5 depicts the MTT assay results that depict the cellular metabolic activity, a critical indicator of cell proliferation rate. The designed PMO-PpIX nanoconjugates exhibited dose-dependent cytotoxicity HT-29 cells in the light-irradiated treatments, which could be attributed to the photochemical reaction process and provoked the ROS generation by PpIX molecules in the presence of light. The bioefficacy of PMO-PpIX treatment was proportionally higher than that of the PpIX treatment at a similar concentration, attributing to the photostability of the PpIX in the post-modified PMO nanospaces.

**Figure 6:**
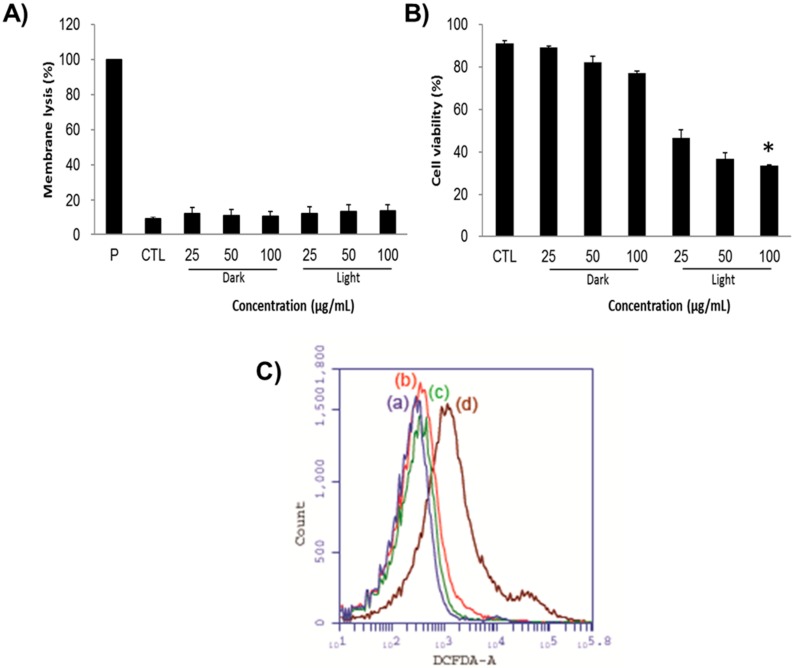
(**A**) Lactate dehydrogenase (LDH) levels representing the cell membrane lysis rate after treating the HT-29 cells with various concentrations of PMO-PpIX (25, 50, and 100 µg/mL) in both dark and light conditions provided. Triton X-100 is used as a positive control (P), which refers to the total amount of LDH in cells. Control treatment (CTL) relates to the amount of LDH released from cells without any treatment. (**B**) Respective cell viability rates during the LDH assay. (* *p* < 0.001). (**C**) The ROS determination using the 2′,7′-dichlorofluorescein diacetate (DCFDA) assay by flow cytometry in various treatments of (**a**) the control treatment group in the absence of light, (**b**) the PMO treatment group in the presence of light, (**c**) the PMO-PpIX treatment in the absence of light, and (**d**) the PMO-PpIX in the presence of light.

Indeed, the LDH assay demonstrates the possible mechanistic way of cancer cell death showing that the cells might have undergone either apoptosis or necrosis in the presence of the nanoconjugates and the light irradiation. Notably, the LDH enzyme is often released concomitantly after when the cell is injured or dead corresponding to the cell membrane damage. The LDH levels can be measured from the collected supernatant of cell suspension following the manufacturer’s instructions. Regardless of the concentration levels of PMO-PpIX and light conditions provided during the PDT (Figure 6A), the cell membrane damage rates of all groups were not higher than 15%, in comparison to positive control (P) Triton X-100 (taken as 100%), demonstrating that the PMO-PpIX nanoconjugates were highly biocompatible and the treated cells could hold their membrane integrity from the unexpected necrotic cell death. Furthermore, the calculated cell viability rates (Figure 6B) of corresponding treatments resulted in the significant reduction in cell count after treating with PMO-PpIX in the presence of light, due to apoptosis. To demonstrate the generation of ROS in the cells, we used 2′,7′-dichlorofluorescein diacetate (DCFDA) assay by flow cytometry, in which the fluorogenic dye DCFDA is transformed into the fluorescent dichlorofluorescein (DCF) in the presence of ROS. As depicted in Figure 6C, it could be observed that the fluorescence levels are higher in the cells with PMO-PpIX in the presence of light over other treatments of the control as well as the designed nanocomposite sample groups in the absence of light. These consequences could be plausibly attributed to the generation of ROS by the PpIX encapsulated PMOs in the presence of light. Together, these results state that ROS determination outcomes could be one of the plausible causes of cell apoptosis.

**Figure 7:**
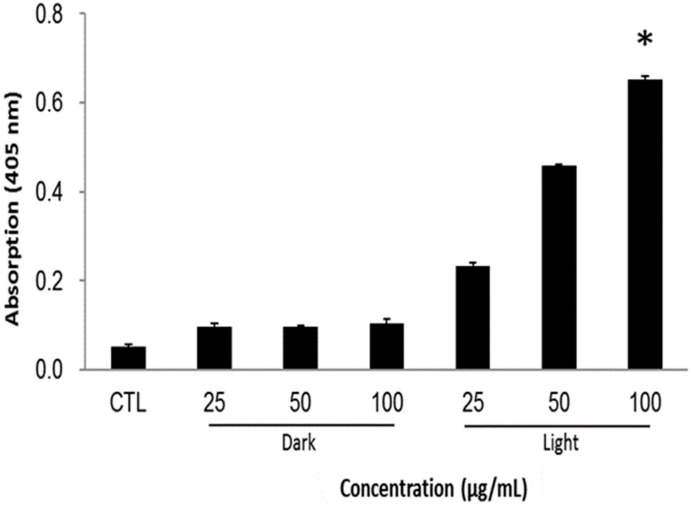
Apoptosis study of HT-29 cells treated with various concentrations (25, 50, and 100 µg/mL) of PMO-PpIX in the absence (**D**) and presence (**L**) of light by the caspase-3 assay. CTL represents a control experiment (cells without any treatment). (* *p* < 0.001).

The resulted cancer cell death can be summarized in a mechanistic way as follows: the singlet oxygen generation after light irradiation alters the cellular mechanisms by, for example, reducing the mitochondrial potential and making the membrane vulnerable for the exchange of cell components. Subsequently, the loss of electrostatic interactions in mitochondria provokes the release of cytochrome *c*, which triggers the apoptotic cascades in the cytoplasm. In the caspases family, caspase-3 plays a crucial role during the cell apoptosis. The caspase levels in cells were measured by using in vitro caspase-3 colorimetric protease assay, whose levels are proportional to the amount of the chromophore released. Figure 7 illustrates that the caspase levels are profoundly higher and dose-dependent in PMO-PpIX treatments in the presence of light than that of the control (CTL) as well as dark treatment (D) groups, demonstrating that PMO-PpIX-treated cells indeed underwent apoptosis pathway and resulted in cell death. To clarify the absorption overlapping of PpIX in the corresponding wavelength region of caspase assay, we validated by recording the absorbance at 405 nm of the supernatant by centrifugation (21,000 *g*) for 17 min, as well as the suspension of the PMO-PpIX at the equivalent concentrations (data not shown). The absorbance value of PpIX in the PMO-PpIX at 405 nm was 3-fold lesser at the highest selected concentration over the absorption of the chromophore generated during the caspase assay, indicating no substantial influence of PpIX in the absorption of chromophore generated in the caspase-3 colorimetric protease assay. On the other hand, the supernatant resulted in deficient absorption, attributing to the stable encapsulation of PpIX guest species on the PMO surfaces, as shown in the stability studies (see Figure 3C). Hence, we believe that these biocompatible and stable PpIX-encapsulated PMOs could be used for light-induced therapeutics efficiently.

### 2.5. Antibacterial Efficacy

**Figure 8:**
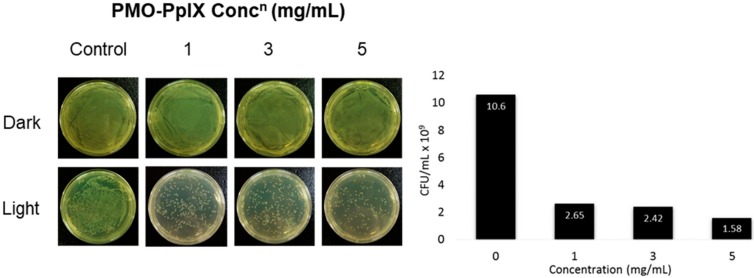
Anti-microbial PDT effect of PMO-PpIX. *E. coli* strain treated with various concentrations of PMO-PpIX in the absence (**D**) and presence (**L**) of green light. Graph showing the colony-forming units (CFUs) count concerning the concentration of PMO-PpIX in the presence of light.

Light-induced microbial inactivation has drawn the increased attention of researchers in the past few decades. Moreover, the upsurge in the antibacterial infection has been a major clinical concern these days. In addition to anticancer efficacy, these designed nanocomposites encapsulated with the positively charged photosensitizer; PpIX molecules were tested towards killing the bacteria. To demonstrate these facts, herein, the antibacterial activity of PMO-PpIX conjugates at various concentrations in the absence (D) and presence (L) of green laser light using Gram-negative bacterium, *Escherichia coli (E. coli)* was assessed using the spread plate method. Figure 8 illustrates that the PMO-PpIX (1-5 mg/mL) sample significantly inhibited the bacterial growth compared to that of the treatment in the dark conditions. The light-assisted PMO-PpIX samples exhibited a dose-dependent reduction in the CFUs of bacteria, and the number of colonies reduced with the increase in the dose of the sample, demonstrating that PMO-PpIX is quite effective in inhibiting *E. coli* growth. Together, these experimental results suggest that the PMO-PpIX sample at a lower concentration and laser light in the visible wavelength region (532 nm) are highly beneficial for PDT. However, further investigations are required to elucidate the antibacterial therapeutic effects comprehensively.

## 3. Materials and Methods

### 3.1. Materials

PpIX, Pluronic F127, 1, 4-bis(triethoxysilyl) benzene (BTEB), potassium chloride (KCl), 1, 3, 5-trimethylbenzene (TMB), Fe(II)SO_4_, and ferric (III) chloride hexahydrate (FeCl_3_. 6H_2_O), 3-aminopropyl triethoxysilane (APTS), potassium phosphate monobasic (KH_2_PO_4_), N, N’-disuccinimidyl carbonate (DSC), diisopropylethylamine, ABMDMA dye, H_2_O_2_, potassium phosphate dibasic (K_2_HPO_4_), MTT, DCFDA, and sodium phosphate dibasic (Na_2_HPO_4_) were obtained from Sigma Ltd. (St. Louis, MO, USA). 

### 3.2. Synthesis of PMOs

The uniform-sized benzene-bridged PMO nanoparticles were fabricated using Pluronic F127 as the surfactant template and BTEB as a silica source in an acidic environment [12,21]. Initially, KCl, TMB, and Pluronic F127 were added to a hydrogen chloride solution (HCl, 2 M, 40 °C). Furthermore, BTEB was dissolved and stirred for 24 h, and then the reaction mixture was subjected to hydrothermal treatment at 100 °C for an additional 24 h. Then, the nanoparticles were collected (12,000 rpm, 17 min), and the surfactant template was extracted by stirring in the acid–ethanol mixture (HCL in ethanol, EtOH) at 70 °C for 12 h. Finally, the particles were collected after several washes with EtOH.

### 3.3. Post-modification of PMOs

Initially, the fabricated benzene-bridged PMOs were functionalized with hydroxyl groups donated by H_2_O_2_ through Fenton and Fenton-like reactions accompanied by electrophilic substitution under appropriate catalytic conditions involving metal precursors (Fe(II)SO_4_ and Fe(III)Cl_3_) and H_2_O_2_ in aqueous sulfuric acid [37]. These metal precursors were added to aqueous sulfuric acid and stirred at 50 °C for 30 min. After complete dissolution, H_2_O_2_ was added and stirred further for 12 h. Then, the residual metal ions were extracted by stirring the modified PMOs in the acid–ethanol mixture for 12 h at 70 °C, and the resultant particles were collected after several washes with EtOH. The samples obtained using Fe(II)SO_4_ and Fe(III)Cl_3_ were denoted as PMO-OH(II) and PMO-OH(III) respectively.

### 3.4. Immobilization of Amine Functional Groups

The designed PMOs were functionalized with amine functional groups to improve the interactions and promote the host–guest chemistry for encapsulating PpIX molecules. The post-modification of PMO-OH (PMO-OH(II) and PMO-OH(III)) with amine functional groups was performed by subsequent addition of designed PMO-OH and APTS in EtOH and stirred for 12 h at 80 °C. The resultant PMO nanoconjugates were collected (12,000 rpm, 17 min) after several washes with EtOH and stored in 99% EtOH. The respective samples were denoted as PMO-OH(II)-NH_2_ and PMO-OH(III)-NH_2_, corresponding to products of Fenton and Fenton-like reactions, respectively. Another batch of surfactant-extracted PMOs was conjugated with amine groups following a similar method discussed above to compare the stability by hydroxylation in PMOs, and this sample was denoted as PMO-NH_2_.

### 3.5. Immobilization of Activated PpIX

Considering the stability studies of various samples of PMOs’, the stable PMO-OH(II)-NH_2_ was chosen, and the drug cargo was loaded in the nanospaces. Initially, PpIX was modified to improve the hydrophilicity and then immobilized in PMO-OH(II)-NH_2_ nanoparticles. The PpIX (44 mg) was first activated by adding DSC (150 mg) and diisopropyl ethyl amine (97 mL) in 20 mL of N, N-dimethylformamide (DMF), and was stirred for 16 h in the dark. Furthermore, 200 mg of PMO-OH(II)-NH_2_ nanoconjugates was added to the activated PpIX solution and stirred at 40 °C for 20 h. Finally, the PpIX-immobilized PMOs were washed twice, each with EtOH and water. The samples were further collected by centrifugation at 21,000× *g* for 17 min, and the pellets were stored after freeze-drying [38]. The sample was shortly denoted as PMO-PpIX. The loading amount of PpIX in PMOs was calculated by measuring the residual amounts of activated PpIX molecules in the supernatant using the UV-vis spectrophotometer (Genequant-1300, GE Healthcare Biosciences, Pittsburgh, PA, USA).

### 3.6. Characterizations

To validate the immobilized functional groups, FT-IR spectra were chronicled on a Bruker Alpha spectrometer (Billerica, MA, USA) using the dried potassium bromide (KBr) pellet method. N_2_ adsorption-desorption isotherms were obtained at 77 K on a Micrometric ASAP 2020 apparatus (Micromeritics, USA) to determine the textural properties. Fluorescence intensity was recorded using the EnSpire Multilabel plate reader (Perkin Elmer Inc., Santa Clara, CA, USA). The zeta potential values corresponding to the surface charge, as well as particle size distribution, were measured by DLS using a Malvern-Zetasizer Nano ZS 90 (ZetaPALS, Malvern Panalytical, Malvern, Worcestershire, UK).

### 3.7. Ninhydrin Assay

Ninhydrin reagent is widely used for the detection of primary amines. In general, the ninhydrin effectively interacts with the terminal amine groups (-NH_2_) in a slightly acidic solution, resulting in a complex with Ruhemann’s purple, which subsequently produces an absorption peak at 570 nm. To validate the immobilized amine functional groups, various PMO nanoconjugates were mixed with an ampule volume of acidic ninhydrin solution. Furthermore, the mixture was heated for 10 min and allowed to cool at room temperature. Before UV-vis measurements, the nanoparticles were removed by centrifugation at 21,000× *g* for 17 min to prevent the background interference. Finally, the UV-vis spectra of the supernatant were recorded.

### 3.8. Stability Assessment

PMOs and its successive nanoconjugates, i.e., modified with NH_2_ functional groups (PMO-NH_2_), and OH functional groups PMO-OH(II) and PMO-OH(III) as well as their successive amines (PMO-OH(II)-NH_2_ and PMO-OH(III)-NH_2_), were subjected to the stability assessment at room temperature. All these samples (1 mg) were suspended well in PBS (1 mL) and allowed to stand for a stipulated time to observe the sedimentation of nanoparticles.

### 3.9. Biocompatibility Study

The hemolysis assay is one of the best toxicity evaluation tests to cross-verify the biocompatibility of nanoparticles in vivo [39]. RBCs from goat blood obtained at Hualien District Agricultural Improvement Station were mixed well with different concentrations of PMO-OH(II)-NH_2_ sample and allowed to stand for 3 h. Furthermore, the supernatant was collected by centrifuging the samples, and its absorbance was recorded at 570 nm. The percentage of hemolysis was then calculated by using the following formula, i.e., (Sample_abs_ − N_abs_)/(P_abs_ − N_abs_) × 100, where N represents negative control (RBCs in PBS), and P represents positive control, (RBCs in dd-H_2_O).

### 3.10. Photobleaching Effect

Indeed, it should be noted that the photosensitizer stability is improved, when immobilized in an appropriate nanocarrier. The effect of light on the stability of the photosensitizer was tested by irradiating the specific light for a given period, and the method is as follows. PMO-PpIX nanoconjugates (10 mg) and equivalent molar concentration of free PpIX were uniformly dispersed in 1.0 mL of dd-H_2_O, and the samples were illuminated with green laser light (532 nm). During the study under long-term laser irradiation, the sample was placed in 4 °C, so that the samples could be cooled down to reduce the thermal effects. The changes in the absorption intensity of PpIX were recorded using the UV-vis spectrophotometer every 10 min for 120 min [40].

### 3.11. Detection of Singlet Oxygen Production

The reagent, ABMDMA, is commonly used to detect singlet oxygen species in vitro by generating the corresponding endoperoxide through bleaching effect. PMO-PpIX nanoconjugates (20 mg) were dispersed in 0.1 mM of ABMDMA in dd-H_2_O, following illumination with green laser light (532 nm) for 6 min, in addition, compared with the control group, ABMDMA alone. The absorption intensity at a wavelength of 400 nm was recorded using the UV-vis spectrophotometer every 30 s during illumination, confirming the singlet oxygen generation.

### 3.12. PDT Efficacy

Notably, the PMO-PpIX nanocomposites were irradiated with green laser light (532 nm, the energy density of ca. 18 J/cm^2^) equipped with a power meter (Ophir NOVA-II) throughout the PDT experiments. Moreover, the respective procedures were followed to determine the bioefficacy of the nanoconjugates. For the PDT experiments, the cells were mainly cultured in a 96-well plate. However, the nanoparticles were treated, and the corresponding wells were irradiated such that adjacent wells on the four sides (left, right, top, and bottom) were left empty to avoid the interference of light with the cells in the corresponding wells. The laser set-up is mainly placed such that the light is placed at the height of 30 cm above the culture plate. Since the light source of laser is a circular point and the light diameter is very similar to the circular well. Therefore, the irradiated light beam can be evenly distributed throughout the well over the cell surface. Indeed, the clinical advantage for the use of red light in the longer wavelength range is that it could offer high tissue penetration; however, decreased thermal cytotoxic effect, preventing the damage of connective tissues of collagen and elastin. Therefore, tissues such as hollow organs could well preserve the mechanical integrity [41]. In this study, we intended to explore the PDT effects of PpIX-based samples in the presence of green light, as PpIX molecules exhibit a strong absorption peak at this wavelength region. Moreover, this equipment is also convenient to handle in the laboratory to illuminate the samples.

#### 3.12.1. Cell Culture

Human colorectal cancer cells (HT-29 cell line) were acquired from the Bioresource Collection and Research Center (Hsinchu, Taiwan). The cell line was subcultured at regular intervals by incubating in Roswell Park Memorial Institute (RPMI)-1640 (GIBCO/BRL Life Technologies, Grand Island, USA) medium added with fetal bovine serum (FBS, 10%, v/v, GIBCO), and 1% antibiotics (penicillin and streptomycin), maintained at 37 °C, 5% CO_2_. The reason behind choosing the HT-29 cell line is that the PMOs’ design, which protects the photosensitizer inside the mesochannels, could be used conveniently for oral administration (as stated in Section 3.12, PDT efficacy). 

#### 3.12.2. MTT Assay

The effects of designed nanoconjugates on the HT-29 cells in the presence of light irradiation were demonstrated using the MTT assay [42]. Indeed, the MTT reagent is used to measure the cell metabolic activity, which could be used as an indicator of cell proliferation rate. Briefly, the HT-29 cells were seeded (1 × 10^4^ cells/well) in a 96-well culture plate by dispersing them in RPMI-1640 medium with 10% FBS. After 24 h, different concentrations of PMO-PpIX nanoconjugates in the FBS-free medium were added to cells and incubated for 4 h to allow the cellular internalization. Herein, the FBS-free medium was used to disperse the PMOs to avoid the unnecessary adsorption of serum protein and nanoparticles, which could result in the aggregates and substantially influence the cellular uptake of the nanoparticles. Later, the corresponding wells were washed with PBS and irradiated with green laser light for 1 min. Then, the cells were nourished with FBS-supplemented medium and incubated further for 20 h. Furthermore, 20 µL of MTT (5 mg/mL in PBS) was added to all the wells and incubated for 4 h. Finally, the pirated wells were added with 150 µL of DMSO to dissolve the water-insoluble violet formazan crystals, and OD values at 570 nm were recorded to calculate the percentage cell viability compared to the negative control.

#### 3.12.3. LDH Assay

The release of the LDH enzyme, often referred to as an apoptosis indicator, was determined based on colorimetric assay in vitro using the TOX-7 LDH-based kit, according to the manufacturer’s instructions (Sigma). LDH is one of the stable cytoplasmic enzymes utilized to correlate its levels with the extent of cell membrane damage after the nanoparticles’ treatment. Briefly, HT-29 cells (1 × 10^6^ cells/well of a 6-well plate) were treated with various concentrations of PMO-PpIX nanoconjugates and incubated for 4 h to allow the particle uptake. In addition, 0.1% Triton X-100 was used as a positive control. Then, the corresponding wells were irradiated with light for 1 min and incubated further for 20 h. Furthermore, the supernatants of all treatments were separated by centrifuging the suspension (250× *g*) for 4 min. Furthermore, the process was continued following the manufacturer’s instructions.

#### 3.12.4. Determination of ROS

The ROS levels were determined using the DCFDA assay following the reported procedure [43]. Briefly, HT-29 cells dispersed in RPMI-1640 medium with 10% FBS were seeded in a 96-well culture plate (1 × 10^4^ cells/well) and incubated for cell attachment. After 24 h, 50 μg/mL of PMO and PMO-PpIX nanoparticle-based samples were prepared in the serum-free medium and incubated with cells for 4 h. After washing the wells twice with PBS, 100 μL of DCFDA (20 μM) was added and incubated for 30 min. Furthermore, the cells were washed twice with PBS, and corresponding treatments were irradiated with a 532 nm green laser light for 1 min. Finally, the harvested cells with the trypsin-EDTA solution were then suspended cells in PBS for flow cytometry measurement at the FL-1 green channel (BD Accuri-C5, Becton Dickinson Biosciences, New Jersey, USA).

#### 3.12.5. Apoptosis

Caspase-3 is one of the important apoptosis cascades, i.e., cysteine-containing aspartate-specific protease, detected through caspase-3 colorimetric protease assay kit (R&D Systems Inc, Minneapolis, USA). The caspase-3 enzyme from the cells interacts with dithiothreitol (DTT), and DEVD-pNA would result in the chromophore p-nitroaniline (pNA), indicating the caspase-3 activity proportional to a number of chromophores. Therefore, the chromophore absorbance values at 405 nm are used to represent caspase-3. Briefly, the HT-29 cells (1 × 10^6^ cells/well of a 6-well plate) were treated with various concentrations of PMO-PpIX nanoconjugates. After 4 h, for PDT effect, the corresponding groups were irradiated with green laser light for 1 min and incubated for an additional 20 h. The cells were then scraped from the plate and damaged the cell membrane by using cold lysis buffer. Furthermore, the supernatants were collected for protein quantitation, which was performed with a fixed proportion of kit reagents, and the absorbance values were eventually measured at 405 nm.

#### 3.12.6. Bacterial Culture

The PDT-assisted inactivation of bacteria was demonstrated using *E. coli* strain through the CFU assay. The bacterial strain was sub-cultured every 1–2 days in the Luria−Bertani (LB) broth medium and incubated in an orbital shaker (37 °C, 150 rpm). Given that most of the outside surface of the cell walls of *E. coli* are composed of the lipid bilayer, we have selected this strain to validate our hydrophobic PMO material as the photosensitizer carrier, which could enrich the affinity between the nanoparticles and the cells.

#### 3.12.7. CFU Assay

The photo-inactivation was measured by calculating the CFUs through the spread plate method [44]. A lawn of bacterial culture was grown overnight in LB broth, and then the cell cycle was restarted by inoculating 100 μL of fresh culture into the LB broth. After 1 h of incubation, aliquots of culture at approximately 1 × 10^8^ CFU/mL were measured at OD_600_. Furthermore, 500 μL of the designed nanoconjugates (PMO-PpIX) of various nanoparticle concentrations in LB broth were incubated with 500 μL of *E. coli* suspension for 4 h in the dark. The corresponding samples were then irradiated using the green laser light (532 nm, the energy density of ca. 18 J/cm^2^) for 1 min along with control groups containing cells alone at 37 °C. Aliquots of each sample were serially diluted and plated in triplicates on Petri plates containing LB agar. After incubation at 37 °C for 24 h, the total number of CFUs was counted.

### 3.13. Statistical Analysis

The data were expressed as mean ± standard deviation (SD) (n = 3) and were analyzed by one-way analysis of variance (ANOVA) followed by a Tukey’s test at a defined level of significance of *P* < 0.001. The experimental data were analyzed using GraphPad Prism (Version 7.0, GraphPad Software, San Diego, CA, USA).

## 4. Conclusions

In summary, we have designed an organo-inorganic nanohybrid platform utilizing PMOs, and its successive modifications enhanced the PDT effects against cancer and bacteria. Upon post-synthesis hydroxylation through electrophilic substitution, these benzene-bridged substantially increased the stability and biocompatibility of PMOs. The resultant Fenton-based PMO nanoconjugates were highly stable and also protected the encapsulated pre-modified PpIX photosensitizer. The incorporation of such photosensitizers in the modified PMOs serves as a potential nano-platform for light-induced cancer therapeutics and effective anti-bacterial modalities. In conclusion, our studies show that the design of post-modified nanocomposite has high advantageous for in vitro PDT in human colon cancer cells.

## Figures and Tables

**Table 1:** Structural properties and Zeta potential values after various modifications in PMOs.

Samples	Surface Area (m^2^/g)	Pore Volume (cm^3^/g)	Pore Size (nm)	Zeta Potential (mV)^a^	Particle Size (nm) ^a^
PMOs	613	2.06	7.23	−6.6	106.1
PMO-OH(II)-NH_2_	635	2.15	7.40	−5.7	94.8
PMO-OH(III)-NH_2_	672	2.21	7.62	−4.9	99.4

^a^ Values are recorded at pH-7.0.
